# Toward New Assessment of Knee Cartilage Degeneration

**DOI:** 10.1177/19476035221144746

**Published:** 2022-12-21

**Authors:** Romain Aubonnet, Jorgelina Ramos, Marco Recenti, Deborah Jacob, Federica Ciliberti, Lorena Guerrini, Magnus K. Gislason, Olafur Sigurjonsson, Mariella Tsirilaki, Halldór Jónsson, Paolo Gargiulo

**Affiliations:** 1 Institute of Biomedical and Neural Engineering, Reykjavik University, Reykjavik, Iceland; 2 Landspitali, University Hospital of Iceland, Reykjavik, Iceland; 3 Medical Faculty, University of Iceland, Reykjavik, Iceland

**Keywords:** image segmentation, knee cartilage, classification, osteoarthritis, 3D models

## Abstract

**Objective:**

Assessment of human joint cartilage is a crucial tool to detect and diagnose pathological conditions. This exploratory study developed a workflow for 3D modeling of cartilage and bone based on multimodal imaging. New evaluation metrics were created and, a unique set of data was gathered from healthy controls and patients with clinically evaluated degeneration or trauma.

**Design:**

We present a novel methodology to evaluate knee bone and cartilage based on features extracted from magnetic resonance imaging (MRI) and computed tomography (CT) data. We developed patient specific 3D models of the tibial, femoral, and patellar bones and cartilages. Forty-seven subjects with a history of degenerative disease, traumatic events, or no symptoms or trauma (control group) were recruited in this study. Ninety-six different measurements were extracted from each knee, 78 2D and 18 3D measurements. We compare the sensitivity of different metrics to classify the cartilage condition and evaluate degeneration.

**Results:**

Selected features extracted show significant difference between the 3 groups. We created a cumulative index of bone properties that demonstrated the importance of bone condition to assess cartilage quality, obtaining the greatest sensitivity on femur within medial and femoropatellar compartments. We were able to classify degeneration with a maximum recall value of 95.9 where feature importance analysis showed a significant contribution of the 3D parameters.

**Conclusion:**

The present work demonstrates the potential for improving sensitivity in cartilage assessment. Indeed, current trends in cartilage research point toward improving treatments and therefore our contribution is a first step toward sensitive and personalized evaluation of cartilage condition.

## Introduction

Osteoarthritis is a prevalent form of arthritis,^1^ happening when protective hyaline cartilage between bones breaks down through injury or disease. Osteoarthritis of the knee is a main reason for impairment, being a significant burden on healthcare systems^2^ with a greater risk to develop with obesity and aging.^3,4^ The study of cartilage thickness is essential to both identify and control the evolution of osteoarthritis. Diagnosis relies on a clinical assessment and a radiographic exam of the joint.^5^ Magnetic resonance imaging (MRI) is the most advanced imaging technique for the evaluation of hyaline cartilage, and presented many improvements in acquisition and image modality in the past years.^5-8^ MRI gives a visual evaluation of the cartilage. Extensive examination of MRI sequences for assessing morphological and structural aspects of knee cartilage are reported in previous studies.^5,6,9^ MRI is able of precisely measuring the thickness of articular cartilage.^7,10,11^ MRI also visualize, other tissues involved in osteoarthritis, such as subchondral bone, meniscus, and soft tissue. It is crucial to understand that osteoarthritis is a disease of the whole organ, involving multiple joint tissues.^5^ Computed tomography (CT) imaging also presents a great 3D representation of cortical bone, osteophytes, and soft tissue calcification. It has been used to study changes in the joint, such as trabecular bone changes, subchondral cysts, and bone sclerosis, that can be osteoarthritis-related alterations in the joint.^12^

Severity of osteoarthritis can be estimated by the grading of joint space narrowing and damage to cartilage and related bone. Different scales exist to evaluate the degree of osteoarthritis. Kellgren-Lawrence grading is used for assessment of osteoarthritis on planar x-rays, where the presence of an osteophyte (Kellgren-Lawrence grade 2) supports a diagnosis of osteoarthritis.^13^ Kellgren-Lawrence relies on joint space narrowing and osteophyte presence to propose a global grade of osteoarthritis, which inaccurately considers that these changes appear continuously.^5^ Another grading system, the Osteoarthritis Research Society International Atlas system, differentiates a joint space narrowing grade from the presence of osteophytes. Yet, they only assess the tibiofemoral joint, reducing the patellofemoral contribution to the disease.^5^ Another widely used scaled is the Ahlbäck^14^ grading which relies on the measurement of joint space narrowing. A study reporting the inter- and intra-observer reliability of the Ahlbäck scale listed low- to medium-agreement coefficients, especially when studying radiographs of earlier stage osteoarthritis.^15^ Observations of knee osteoarthritis scales revealed moderate correlation with arthroscopic findings and moderate to high reliability between individual observers.^16^ In a study on severe osteoarthritis, 5 radiological grading systems indicated medium correlation with intra-operative findings of full-thickness cartilage loss, and moderate inter-observer reliability for all systems.^17^ In both studies, Kellgren-Lawrence and Ahlbäck showed the highest correlation with cartilage loss, although still the moderate range. Semi-quantitative MRI-based grading systems, Whole Organ Magnetic Resonance Imaging Score (WORMS) and Knee Osteoarthritis Scoring System (KOSS) for instance, are based on a wide range of features of the MR image from the whole knee joint, such as cartilage size and depth, bone marrow lesions, and subchondral cysts. Some of these scoring systems have indicated “within grade” alterations over time, thus showing a higher sensitivity compared to traditional grading systems.^5^ Injury to the knee joint is potential risk of development of osteoarthritis. X-ray imaging, CT, and MRI have all been used in the evaluation of knee trauma consecutive of aninjury.^18^ An MRI-based score integrating traumatic and following degenerative changes was presented in 2014 by Roemer *et al.*^19^ The Anterior Cruciate Ligament OsteoArthritis Score (ACLOAS) evaluates joint damage, features of osteoarthritis (including cartilage loss) and signs of inflammation in traumatic injury to the knee. The ACLOAS aims to be a tool for long-term evaluation of injury and subsequent osteoarthritis in the knee joint. MR-based morphometry for the 3D assessment of cartilage in injury has been performed but most of the work published in this area has been on knees with established osteoarthritis.

Machine Learning (ML) and Deep Learning (DL) are widely used in different bio-medical applications, including medical image analysis. In the scientific literature, there are several applications of ML and DL for MRI of the knee.^20,21^ Liu *et al.*^22^ used DL to detect cartilage degeneration and acute cartilage injuries within the knee joint while Bien *et al.*^23^ developed an efficient DL method to detect general abnormalities and specific diagnosis on MRI exams. DL was also applied for osteoarthritis diagnosis^24^ and to predict patients at high risk of total knee replacement to prevent the surgery with an early-stage diagnosis using both MRI and non-image features.^25^ Kwon *et al.*^26^ used gait data and radiographic images to multi-classify the severity of osteoarthritis based on the Kellgren–Lawrence grade system using DL. Different ML algorithms using MRI as an input are also applied to predict the progression of osteoarthritis using a principal component analysis (PCA) approach on the extracted features^27^ or using plain radiographs and clinical data.^28^ The main scope of ML technologies in this research is to study and understand the predictive potential of the features elaborated from the image analysis and their ability in distinguish degenerative, traumatic, and healthy subjects, with a focus on the features that contribute the most in the classification process. This study compares different cartilage assessment metrics, developing a novel workflow to 3D model bone and cartilage and, moreover, analyzing new features for a more sensitive cartilage assessment, currently required as a support element toward more patient-specific treatment development.

## Material and Methods

**Figure 1.**
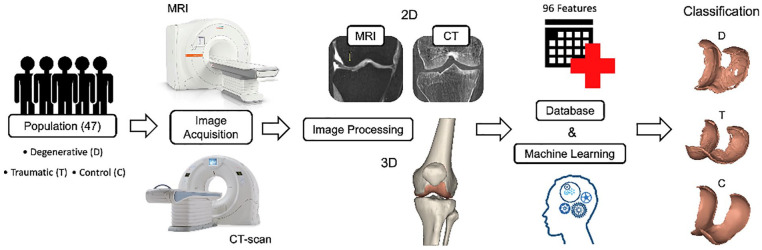
Graphical abstract.

Figure 1 shows the work done in this manuscript starting from the recruitment to the data acquisition, analysis, and computation of the feature importance.

### Participants

Participants were recruited as part of the European project RESTORE (https://restoreproject.eu/) (EU’s Horizon 2020 research and innovation program, grant agreement ID: 814558), whose objective is to develop solutions for personalized cartilage regeneration. This study has been approved by the Icelandic Bioethics Commission (approval number: VSN-19-050). The aim of our research group is to develop a database of morphometric chondral lesions with associated 3D models (clDB). The function of the clDB is to provide accurate 3D models of chondral status, bones, and soft tissue to develop, design, test, and validate 3D printed microtissues that can fit patient-specific lesions.

Recruitment: After completing a written informed consent, 47 subjects (24 females, 23 males, age = 50 ± 19 years) underwent CT and MRI scans of a single knee at Landspitali University Hospital in Reykjavik, Iceland, using standardized acquisition protocols and patient positioning. From the total of patients, 23 subjects (12 females, 11 males, age = 64 ± 12 years) were suffering from degenerative (D) cartilage. They were examined by an orthopedic doctor due to pain from osteoarthrosis and were placed on the waiting list for treatment with total knee arthroplasty (TKA). Sixteen (9 females, 7 males, age = 35 ± 11 years) suffered from a knee trauma (T) with possible cartilage injury. The emergency clinic provided an alert when there is a patient with suspected ligament injury and patella dislocation. They underwent plain x-ray to exclude fracture. Then, they were called to exclude any history of knee injuries or problems. The alert was received within a week, and the patients underwent CT and MRI during the second week from the day of the trauma. Finally, 8 subjects (3 females, 5 males, age = 34 ± 14 years) were involved in the study as control (C) subjects (no symptoms of history of knee trauma/degeneration). For D and T group, in addition to the CT and MRI data that were acquired for this study, X-ray data were also available, as a part of the routine clinical evaluation detailed above. The X-rays were not performed for the C group. **Table 1** sums up the demographics of the patients.

**Table 1.** Description of the Patients Demographics (Age, Gender) by Group.

Category	Degenerative	Traumatic	Control
# Female	12	9	3
Mean age (Std)	66 (12)	39 (11)	29 (5)
# Male	11	7	5
Mean age (Std)	66 (7)	29 (7)	37 (16)

Acquisition protocol: Both protocols were performed with the knee in the same fixated position, evaluated by 2 radiological technicians, and under the supervision of a radiologist.

The CT scanner was a Toshiba Aquillion One, 320 slice, that covered a 16 cm area of interest in a single gantry rotation. Slice thickness was 0.5 mm with an increment of 0.25 mm. Tube voltage was 120 kV, tube current was 250 mA, and effective mAs was 125. The protocol covered about 15 cm of area (axial plane) centered at the knee joint with small variations according to patient size. No intravenous contrast was administered. The preliminary CT dose index (CTDIvol) was set to 12.1 mGy. The preliminary dose-length product was (DLP) 193.2 mGy*cm. These values were individually recalculated by the CT scanner for each patient according to size/thickness of the examined area. The MRI was a 3T Siemens Healthcare Prisma scanner. Volumetric 3D sequences with isotropic voxels of 0.6 mm were acquired in the axial plane with a surface coil without the use of intravenous contrast. This allowed for reconstructions in various planes along regions of interest. A 3D FSE (fast spin echo), intermediate weighted and fat suppressed sequence which allowed for morphologic evaluation of cartilage but also for better assessment of subchondral bone marrow was used. The maximum field of view was 16 cm, with a minimum matrix size of 256 x 256. The area of interest was the cartilage covered areas around the knee. The protocol covered 14 cm centered at the knee joint. The radiographers use a splint to potion the knee on the scanning tables ensuring that the orientation is identical between scans. Scans are taken consecutively.

### 2D Measurements

An exhaustive radiological examination was performed on the bones and articular cartilages of the knee joint to assess their condition. These observations were based on 3 types of 2D medical imaging: X-ray, CT and MRI. The assessment was done on femur and tibia from both the medial (MC) and lateral (LC) compartments as well as on the patella and femoral trochlea within the femoropatellar compartment (FPC) of the scanned knee.

#### Bone

**Figure 2.**
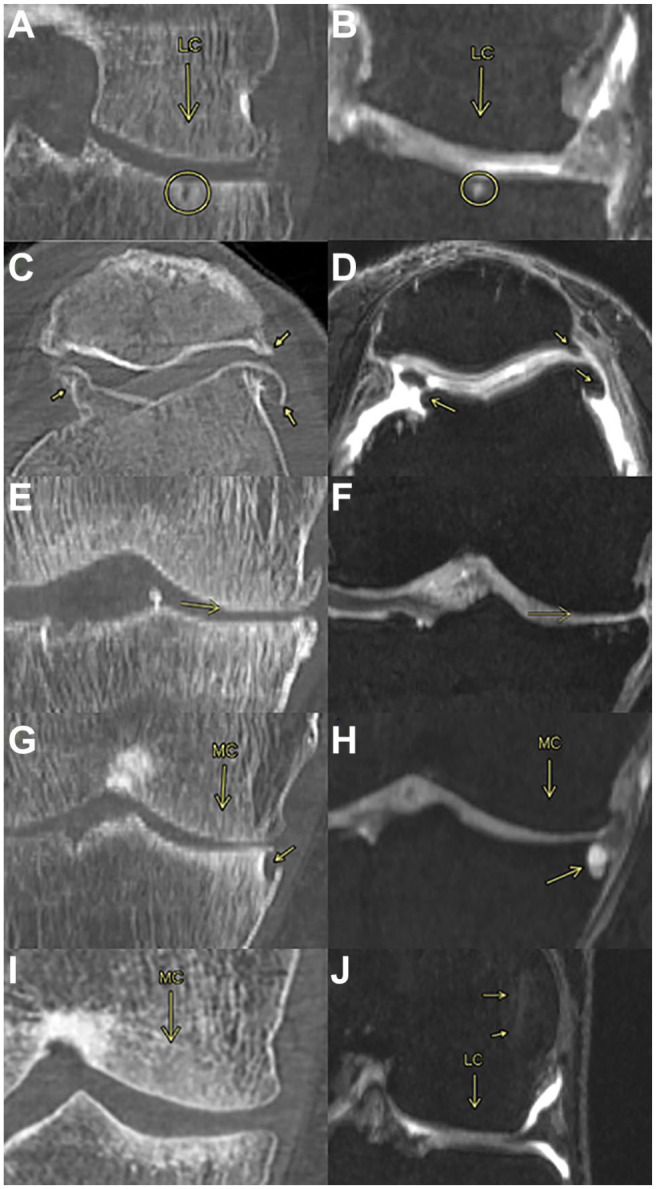
Bone observations. (**A**, **B**) Subchondral bone cysts are typically spherical or ellipsoidal fluid-filled cavities within the subchondral bone region. (**C**, **D**) Osteophytes are cartilage-capped bony proliferations (spurs) that most commonly develop at the margins of a synovial joint as a response to articular cartilage damage. (**E**, **F**) Bone attrition is the result of flattening or depression of the articular surfaces, probably because of bone remodeling. (**G**, **H**) Osteonecrosis is a generic term referring to the ischemic death of the constituents of the bone and is observed as if the bone is missing a piece. (**I**) Subchondral bone sclerosis is a thickening of the bone seen in joints affected by OA. It is observed as a “whitening” of the bone only in CT. (**J**) Subchondral bone edema is a build-up of fluid in the bone marrow as a response to an injury or OA condition visible on MRI but not on CT. MRI = magnetic resonance imaging; OA = osteoarthritis; CT = computed tomography.

**Figure 2** sums up examples and brief definitions of the pathologies observed on the femur, tibia, and patella. The subfigures on the left (A, C, E, G and I) correspond to CT scans while the right subfigures (B, D, F, H, and J) correspond to MRI scans. As observed in **Figure 2**, some pathologies could be found in both CT and MRI, while others (I, J) had a particular 2D image.

#### Cartilage and joint space

**Figure 3.**
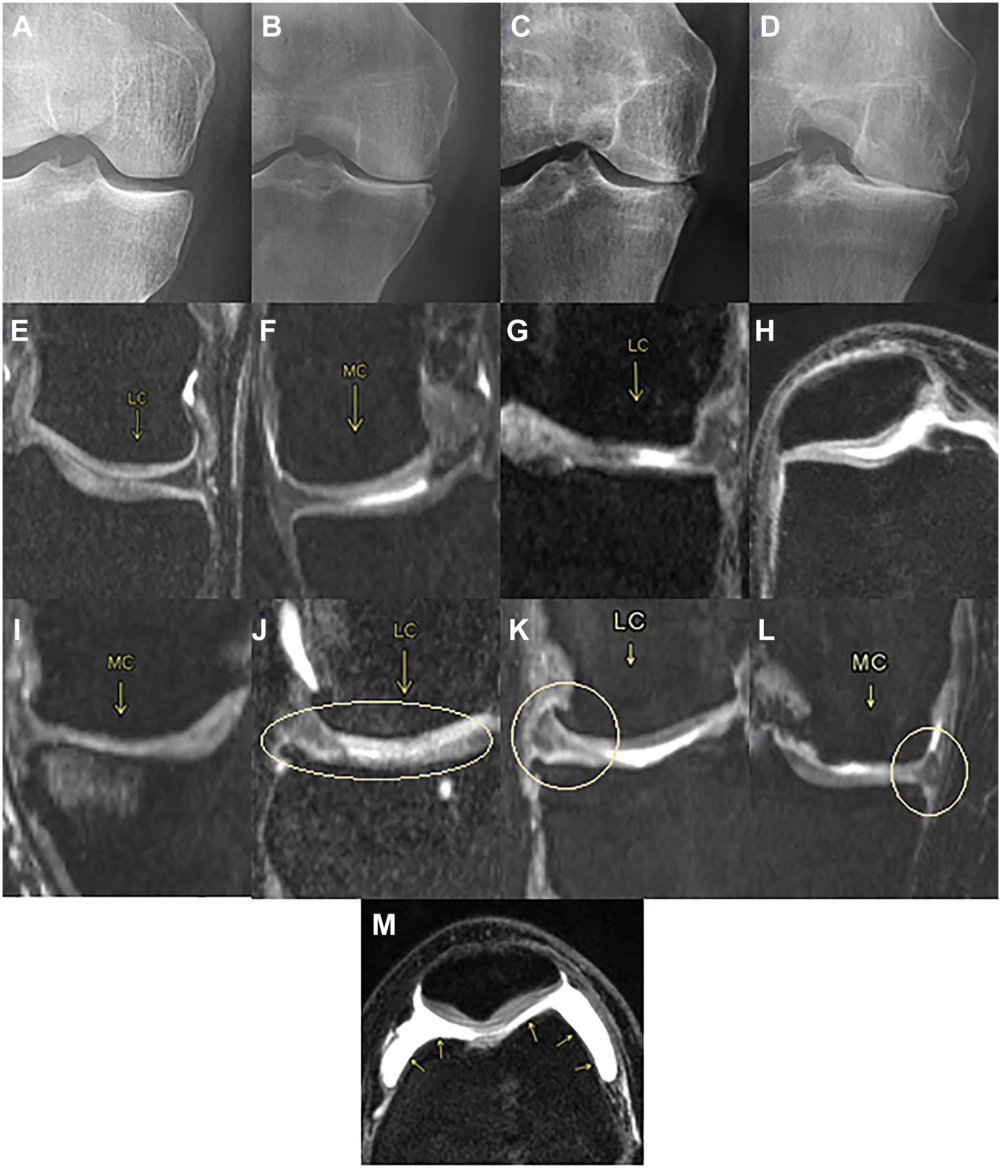
Cartilage and joint space observations. (**A**, **B**, **C**, **D**) Ahlbäck grading is a classification system that focuses on the reduction of the joint space as an indirect sign of cartilage loss. (**A**) grade 0: normal. (**B**) grade 1: joint space narrowing (less than 3 mm). (**C**) grade 2: joint space obliteration (elimination). (**D**) grade 3: minor bone attrition (0-5 mm). (**E**, **F**, **G**, **H**, **I**) ICRS (International Cartilage Repair Society) grading is the most used score system for quantification of existing cartilage defects at the knee. (**E**) grade 0: normal cartilage. (**F**) grade 1: nearly normal cartilage. Superficial lesions; soft indentation and/or superficial fissures and cracks. (**G**) grade 2: abnormal cartilage. Lesions extending down to <50% of cartilage depth. (**H**) grade 3: severely abnormal cartilage. Defects extending down to >50% of cartilage depth; down to calcified layer but not through the subchondral bone. Blisters. Defects more visible toward the medial area of the patella. (**I**) grade 4: severely abnormal. Lesions extending down through the subchondral bone. (**J**, **K**, **L**) Meniscal pathology is associated with an elevated prevalence of MRI-detected cartilage damage. There are 3 types of pathology; (**J**) degeneration: not acute as a tear, this injury is a more gradual onset and tends to occur as we get older. (**K**) rupture: is a tear in the lateral or medial meniscus due to rotational forces directed to a flexed knee. (**L**) protrusion: when the location of the outer edge of a meniscus is beyond the tibial articular surface. (**M**) Synovitis—Effusion. While synovitis is the inflammation of the synovium; effusion is when excess synovial fluid accumulates in or around the knee joint. It is observed generally in the FPC as a white stain. MRI = magnetic resonance imaging.

**Figure 3** contains examples and brief definitions of the gold standard observations made on the cartilage and the joint space. Except for the Ahlbäck grading, which was observed on an X-ray, the rest of the observations were made on MRI scans.

#### Articular cartilage thickness

To manually measure the articular cartilage thickness (ACT) from an MRI scan of the knee, a single investigator followed the methods previously proposed by Koo *et al.*^29^ to segment both femoral condyles into 3 regions of interest. These regions, called anterior, medial, and posterior were estimated as the main weight bearing regions of the articular cartilage in a sagittal view plane.

**Figure 4.**
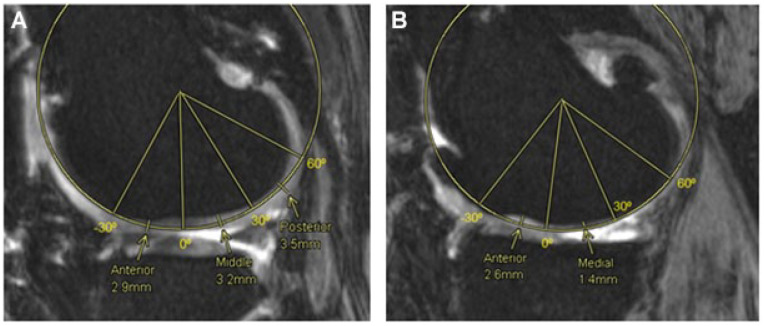
Femoral cartilage thickness measurements, medial and lateral compartments. Fitting cylinder method to obtain 3 regions of interest, anterior (-30º-0º), medial (0º-30º) and posterior (30º-60º) in the lateral compartment (**A**) and the medial compartment (**B**).

The method to define these regions consisted of locating a central axis perpendicular to the sagittal plane by fitting a cylinder that best represented the articular cartilage geometries.^7^ Then, tibiofemoral contact points were identified, and lines were drawn from that central axis to these contact points to define the 3 regions. In the LC, the 0º is defined as the most inferior point of the condyle, while for the MC this point occurs about 20º anterior to that of the LC. The anterior region in both compartments extends 30º anterior to the 0º, the middle region from 0º to 30º posterior to the 0º line and the posterior region from 30º to 60º posterior to the 0º line. Finally, the points where the measurements were taken were located at the center of each of the 3 regions in each compartment, as shown below in **Figure 4**. The values of the ACT shown in the Results section are an average of the 3 contact points (anterior, medial, and posterior) for both medial and lateral compartments.

**Figure 5.**
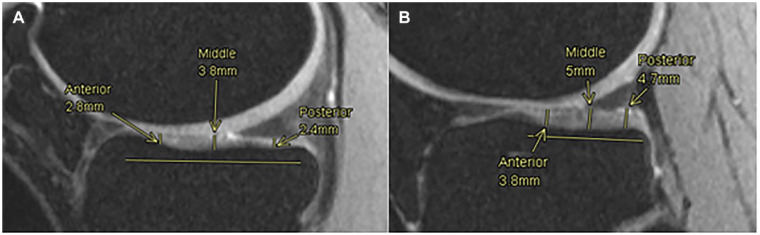
Tibial cartilage thickness measurements. Anterior, middle and posterior points are measured along the tibial cartilage in medial (**A**) and lateral (**B**) compartments.

From the same slices used to measure the femoral ACT in the medial and lateral compartments, the tibial ACT was measured. In both cases and as shown in **Figure 5**, an anteroposterior line representing the length of the tibial cartilage was drawn and divided into 3 regions of equal length. The center of each region (anterior, middle, and posterior) was determined as the point where the cartilage thickness was measured. The values for the tibial cartilage thickness in each compartment are later displayed as the average of these 3 points.

**Figure 6.**
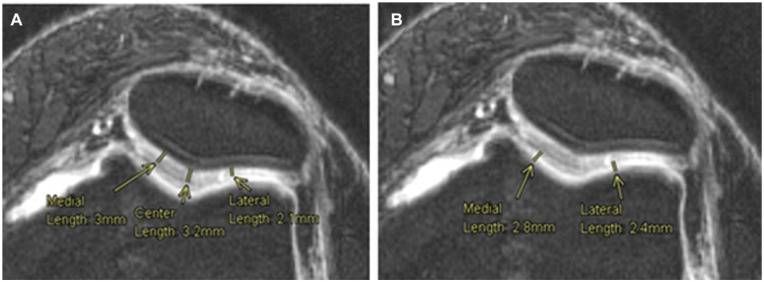
Cartilage thickness measurements, femoropatellar compartment. Measurement of the articular cartilage thickness at 3 points on the patella (**A**) and articular cartilage thickness of the femoral trochlea on 2 different points (**B**).

As for the FPC, the measurements were taken from an axial plane view, where both articular cartilages from the femoral trochlea and the patella were the thickest in the same slice. This means that the chosen slice depended on both cartilages and was not the same for all patients. On the patella, 3 points were used to measure the cartilage thickness, the center one as the most inferior point of the patella, then a medial point as the center point of the medial part of the patella cartilage and a lateral one as the center of the lateral part of the cartilage. Lastly, on the femoral trochlea, 2 points were used to assess the cartilage thickness, 1 at the center of the medial condyle cartilage and another at the center of the lateral condyle cartilage (**Fig. 6**).

#### Cumulative index based on bone conditions

A cumulative index (CI) from 0 to 6 was used to quantify the bone anomalies present in each bone, that is, subchondral cysts, subchondral sclerosis, osteophytes, bone attrition, osteonecrosis, and/or subchondral edema, regardless of the compartment for each patient. Hence, for an observed pathology (either in CT or MRI) a 1 was assigned and consequently, the index was the sum of pathologies present in a certain bone of a certain patient. Sometimes, when the CI is compared against observations made within a compartment such as the Ahlbäck grading (AG) or the International Cartilage Repair Society (ICRS) score, the index is then considered for the bone in question within a compartment.

### 3D Measurements

**Figure 7.**
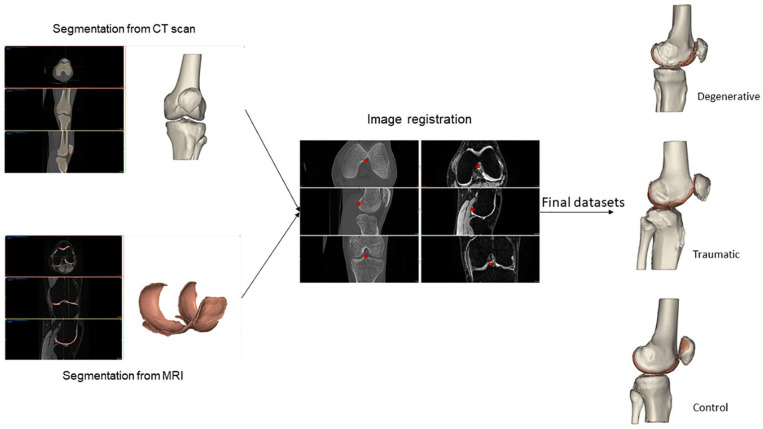
Segmentation workflow.

The DICOM images were imported into a medical device software (MIMICS, Materialize, Belgium). **Figure 7** describes the processing workflow. This workflow was repeated and evaluated 3 times by 3 biomedical engineers, under the supervision of a project manager, to obtain the most accurate segmentation.

The bones (femur, tibia, patella, and fibula) were segmented from the CT scan. From the MRI datasets, the cartilage of femur, tibia, and patella were segmented using the same pipeline: first, a mask was created by setting a density threshold interval, to delimit the part of interest. Then, a manual adjustment was performed to define as precisely as possible the mask for each entity. A visual inspection was done to check that no mask was overlapping with another. Each mask was then calculated in 3D and was smoothed and wrapped to ensure a better model quality. From the CT scans, 4 objects were 3D calculated: the femur, the tibia, the patella, and the fibula (all bones). From the MRI, 4 objects were also 3D calculated: the femoral cartilage, the medial tibia cartilage, the lateral tibia cartilage, and the patellar cartilage.

**Figure 8.**
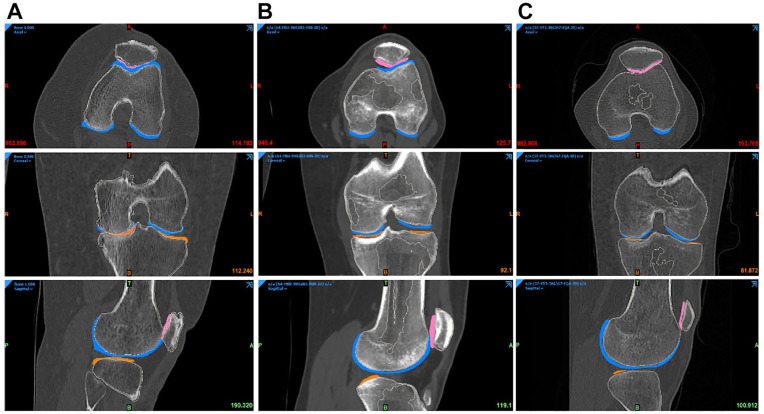
Final registration for the 3 groups of patients (**A**) Degenerative (**B**) Traumatic (**C**) Control.

**Figure 9.**
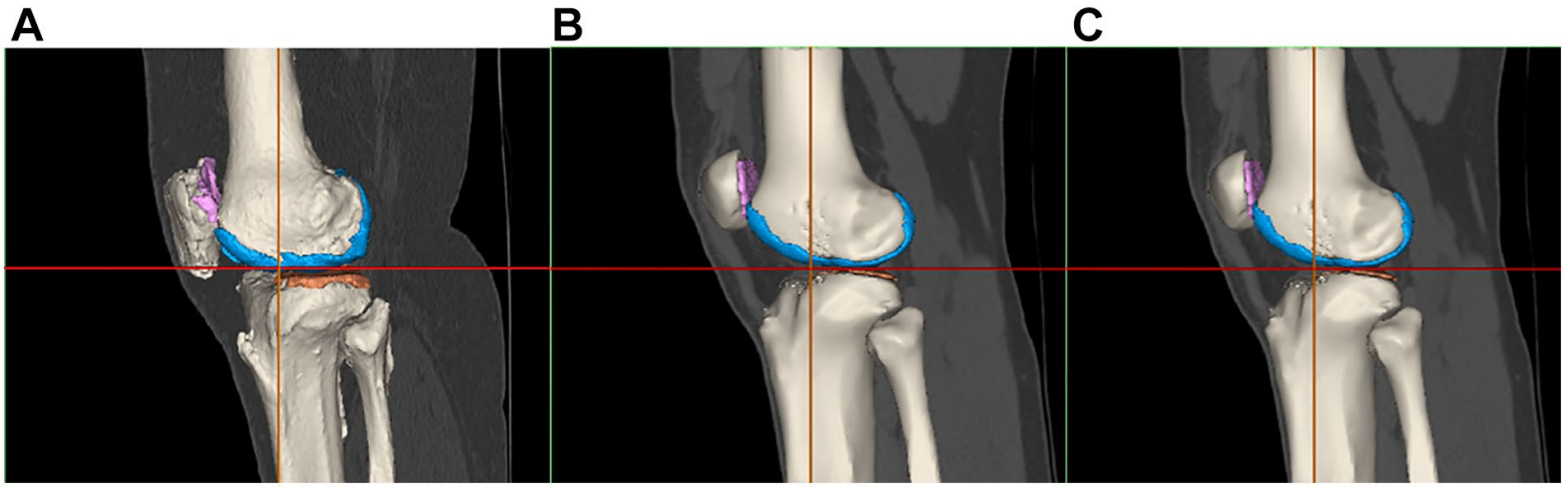
3D model from the registration for the 3 groups of patients (**A**) Degenerative (**B**) Traumatic (**C**) Control.

The next step was the image registration, done from the CT scan. The MRI objects were combined with the CT objects. Bone anatomical landmarks were identified from the 2 scans. The 2 sets can be aligned, by superimposing these points into the 2 separate scans (**Fig. 8**). From the combined dataset, 3D models of femoral, tibial, patella, and fibula bones and cartilages were created and displayed (**Fig. 9**). A visual inspection was done to check that no parts were overlapping, and that the anatomy was respected. If not, manual adjustments or a new image registration were performed.

From this final file, the radiodensity of each part (bone and cartilage), was extracted in Hounsfield Units (HU). The Hounsfield scale is a quantitative scale to define radiodensity. Water is arbitrarily assigned as 0 HU, meaning that materials denser than water have positive values and materials less dense have negative values. The bone mineral density (BMD) (in g/cm3) was computed from the radiodensity using a linear formula (ρ_app_ = 0.000494 · HU + 1.1 [g/cm^3^]) that was determined empirically based on phantoms.^30^

To avoid partial volume effect between 2 shades of gray, an erosion of 1 pixel was performed for each cartilage segment. Next, the cartilage mask was filtered between 0 and 300 HU to eliminate pixels with intensities outside the soft tissues range. The cartilage radiodensity was extracted from this final mask. The volume (in mm^3^) and the surface (in mm^2^) were also computed from each 3D object.

### Data Analysis

#### Statistics

A power analysis is performed to determine if the number of samples in each group is sufficient to have a test with a power of 80% and significance level at 5%. The groups are compared in pairs (D vs T, D vs C, T vs C).

To determine whether there were significant differences between the 3 groups (D, T, C) an analysis of variance (ANOVA) test was performed, and differences were considered significant at *P* < 0.05. If differences were encountered, a *post-hoc-* test with Bonferroni correction was used to determine which group or groups were significantly different.

#### Machine learning

Knime analytics platform (v. 4.3.1)^31^ was adopted to develop the steps of the ML analysis: this software was previously employed in multiple biomedical studies that confirm its efficiency.^32-34^ In the present ML analysis, 2 tree-based algorithms were applied to the multi-classification of the degenerative, traumatic, and healthy (control) patients: random forest (RF) and gradient boosting (GB).^35,36^ Both RF and GB rely on the use of the decision tree creating an ensemble of trees which drastically increases the prediction performance and addresses the instability of a single tree.^37^ The tree-based algorithms are demonstrated to be efficient if applied to biomedical datasets,^38^ as also discussed in the Cambridge University published book by Malley *et al.*,^39^ and, in general, clinicians can more easily appreciate them as they give a very human representation of the data.^40^ RF works by combining randomization and bagging: it builds a set of basic decision trees during the training and the predicted class is the mode of the classes of the individual tree. GB applies boosting and randomization in a similar way to RF, adding bagging techniques that assign a higher weight to wrongly classified. RF was performed using the same random seed for every model and the same hyper-parameters (number of trees = 100, split criterion = Information Gain Ratio, maximum 3 depth = 10, and minimum node size = 1). The same was done with GB (number of trees=100, maximum 3 depth = 4, and learning rate = 0.1). As reported in Kohavi^41^ and widely used in literature,^42,43^ the 10-fold cross validation was performed for the train and test division of the dataset to have a complete and reliable view of all the dataset during the ML analysis. Accuracy, precision, recall, and F1 have been considered as classification metrics.^44^ Accuracy (equation [1]) is defined as the number of correct predictions divided by the total number of predictions. Precision (equation [2]) is the meare of patients that we correctly identify as having the disease out of all the patients having it, while for all the patients who have the disease, recall (equation [3]) tells us how many the algorithm correctly identified as having the disease. F1 (equation [4]) is defined as the harmonic mean between precision and recall. The respective equations with True Positive (TP), True Negative (TN), False Positive (FP), and False Negative (FN) nomenclature are as follows.

(1) $$\mathrm{Accuracy}=\frac{TP+TN}{TP+TN+FP+FN}$$

(2) $$\mathrm{Precision}=\frac{TP}{TP+FP}$$

(3) $$\mathrm{Recall}=\frac{TP}{TP+FN}$$

(4) $$F1=\frac{TP}{TP+\frac{FP+FN}{2}}.$$

Recall, precision and F1 are computed for all the 3 classes to assess the performance of each classification model. To better understand the prediction ability of the different features extracted from the medical image analysis, seven subsets are chosen as input to the tree-based algorithms: details can be seen in **Table 2**. Based on the ML models with the best results for each subset, a feature importance analysis is performed using the software tools included in Knime analytics platform (v. 4.3.1).^31^

**Table 2.** Feature Selection Sets Used as Inputs for the ML Analysis.

		Source
Tot Feat	96	All the available features from MRI, CT, and 3D elaboration
2D Feat	78	Features from MRI (52) and CT (26)
3D Feat	18	Features of cartilage volume and density (and its standard deviation) from 3D elaboration
Ct—Scan Feat	26	Features from CT—part of the 2D group
MRI Feat	52	Features from MRI—part of the 2D group
Bone Feat	50	Features of Bone from CT and MRI (subchondral bone cysts, sclerosis and edema, osteophytes, osteonecrosis, and bone attrition)
Cartilage Feat	26	Features of Cartilage from MRI (ICRS grades, meniscal pathology, synovitis-effusion, and measurements of thickness)

ML = machine learning; MRI = magnetic resonance imaging; CT = computed tomography; ICRS = International Cartilage Repair Society.

## Results

As stated in the Methods section, the aim of our research group in RESTORE project is to develop a database of morphometric chondral lesions with associated 3D models (clDB). The database provides a benchmark for 3D bioprinting design and to advance cartilage assessment. The database is open access and available at: https://restore-project.ru.is/

### 2D Measurements

The results shown in **Table 3** display a summary of the pathologies observed in bone, the meniscus and the synovitis-effusion as percentages for each group of patients.

**Table 3.** 2D Measurements Results. Summary of Bone Pathologies, Meniscal Pathology and Synovitis-Effusion as Percentages for Each Group of Patients.

Pathology	Compartment/Bone	Degenerative	Traumatic	Control
Subchondral bone cysts	Medial/femur	34.78%	18.75%	0%
	Medial/tibia	60.87%	6.25%	0%
	Lateral/femur	17.39%	6.25%	0%
	Lateral/tibia	26.09%	12.5%	0%
	Femoropatellar/fem. trochlea	26.09%	18.75%	12.5%
	Femoropatellar/patella	30.43%	6.25%	12.5%
Osteophytes	Medial	91.3%	6.25%	12.5%
	Lateral	95.65%	0%	0%
	Femoropatellar	91.3%	25%	25%
Bone Attrition	Medial/femur	30.43%	0%	0%
	Medial/tibia	26.09%	0%	0%
	Lateral/femur	0%	0%	0%
	Lateral/tibia	0%	0%	0%
	Femoropatellar/fem. trochlea	0%	0%	0%
	Femoropatellar/patella	0%	0%	0%
Osteonecrosis	Medial/femur	4.35%	25%	0%
	Medial/tibia	17.39%	12.5%	0%
	Lateral/femur	21.74%	37.5%	12.5%
	Lateral/tibia	21.74%	25%	12.5%
Subchondral bone sclerosis	Medial/femur	86.96%	37.5%	12.5%
	Medial/tibia	100%	97.35%	87.5%
	Lateral/femur	34.78%	31.25%	25%
	Lateral/tibia	21.74%	12.5%	0%
	Femoropatellar/fem. trochlea	13.04%	6.25%	0%
	Femoropatellar/patella	69.57%	93.75%	75%
Subchondral bone edema	Medial/femur	52.27%	31.25%	25%
	Medial/tibia	65.22%	12.5%	0%
	Lateral/femur	26.09%	68.75%	25%
	Lateral/tibia	13.04%	6.25%	0%
	Femoropatellar/fem. trochlea	17.39%	50%	0%
	Femoropatellar/patella	4.35%	50%	0%
Meniscal pathology	Medial	100%	18.75%	0%
	Lateral	26.09%	37.5%	0%
Synovitis—Effusion		95.65%	100%	75%

The results shown in **Table 4** display the average and standard deviation of the ICRS, cumulative index, and ACT for each group of patients.

**Table 4.** 2D Measurements Results. Average Values of the ICRS Grading, CI and ACT for Each Group (and Standard Deviation Between Parentheses) and Their Location (Compartment/Bone).

Pathology	Compartment/Bone	Degenerative Average (SD)	Traumatic Average (SD)	Control Average (SD)
ICRS	Medial/femur	3.26 (0.67)	1 (1.41)	0.63 (0.70)
	Medial/tibia	3.17 (1.31)	1.13 (1.05)	0 (0)
	Lateral/femur	1.96 (1.37)	1.63 (1.11)	1.38 (0.70)
	Lateral/tibia	2.22 (1.41)	1.75 (1.25)	0.75 (0.83)
	Femoropatellar/fem. trochlea	2.36 (1.37)	0.94 (1.09)	0.75 (1.09)
	Femoropatellar/patella	3.09 (0.79)	2.69 (0.58)	1.75 (0.66)
Cumulative Index	Femur	3.83 (1.19)	2.38 (0.96)	1.13 (0.83)
	Tibia	4.09 (1.24)	1.69 (0.79)	1.25 (0.46)
	Patella	2.04 (0.93)	1.75 (0.93)	1.13 (0.83)
Articular Cartilage Thickness	Medial/femur	1.5 (0.71)	2.63 (0.56)	2.64 (0.67)
	Medial/tibia	1.63 (0.76)	2.3 (0.45)	2.48 (0.42)
	Lateral/femur	2.44 (0.63)	2.7 (0.54)	2.9 (0.38)
	Lateral/tibia	2.4 (0.81)	2.9 (0.73)	3.06 (0.65)
	Femoropatellar/fem. trochlea	2.21 (0.77)	1.97 (0.77)	2.31 (0.29)
	Femoropatellar/patella	2.29 (0.61)	2.78 (0.7)	2.7 (0.32)

ICRS = International Cartilage Repair Society; CI = cumulative index; ACT = articular cartilage thickness.

#### Bone

**Figure 10.**
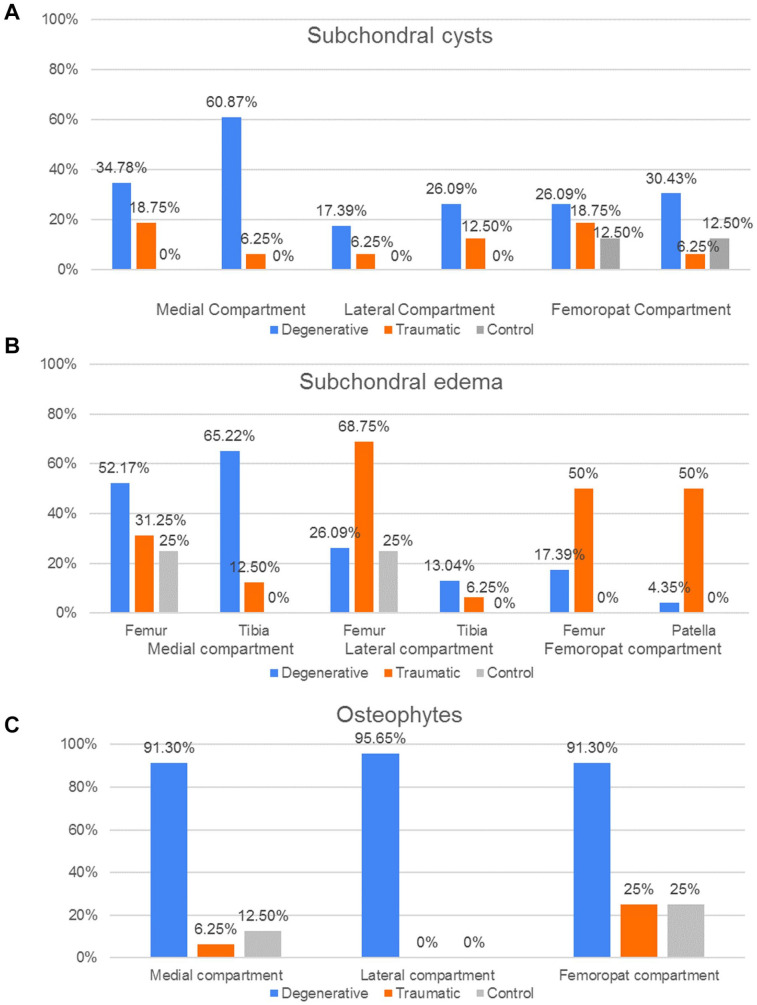
Bone pathologies distribution. Percentages of patient groups (D, T, C) with (**A**) Subchondral cysts. (**B**) Subchondral edema, and (**C**) Osteophytes. according to the respective compartment.

Of the 6 pathologies observed in bone, **Figure 10** only shows the percentages of 3 of them that best indicate differences between the groups. The results with the other pathologies are shown in the appendix.

#### Cartilage and joint space

**Figure 11.**
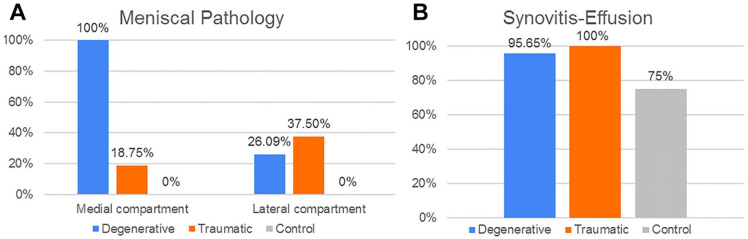
Cartilage and joint space pathology distribution. Percentage of patient groups (D, T, C) with (**A**) meniscal pathology (**B**) synovitis-effusion, according to the respective compartment.

**Figure 11** shows the percentage of patients in each group which presented meniscal pathology and synovitis-effusion.

**Figure 12.**
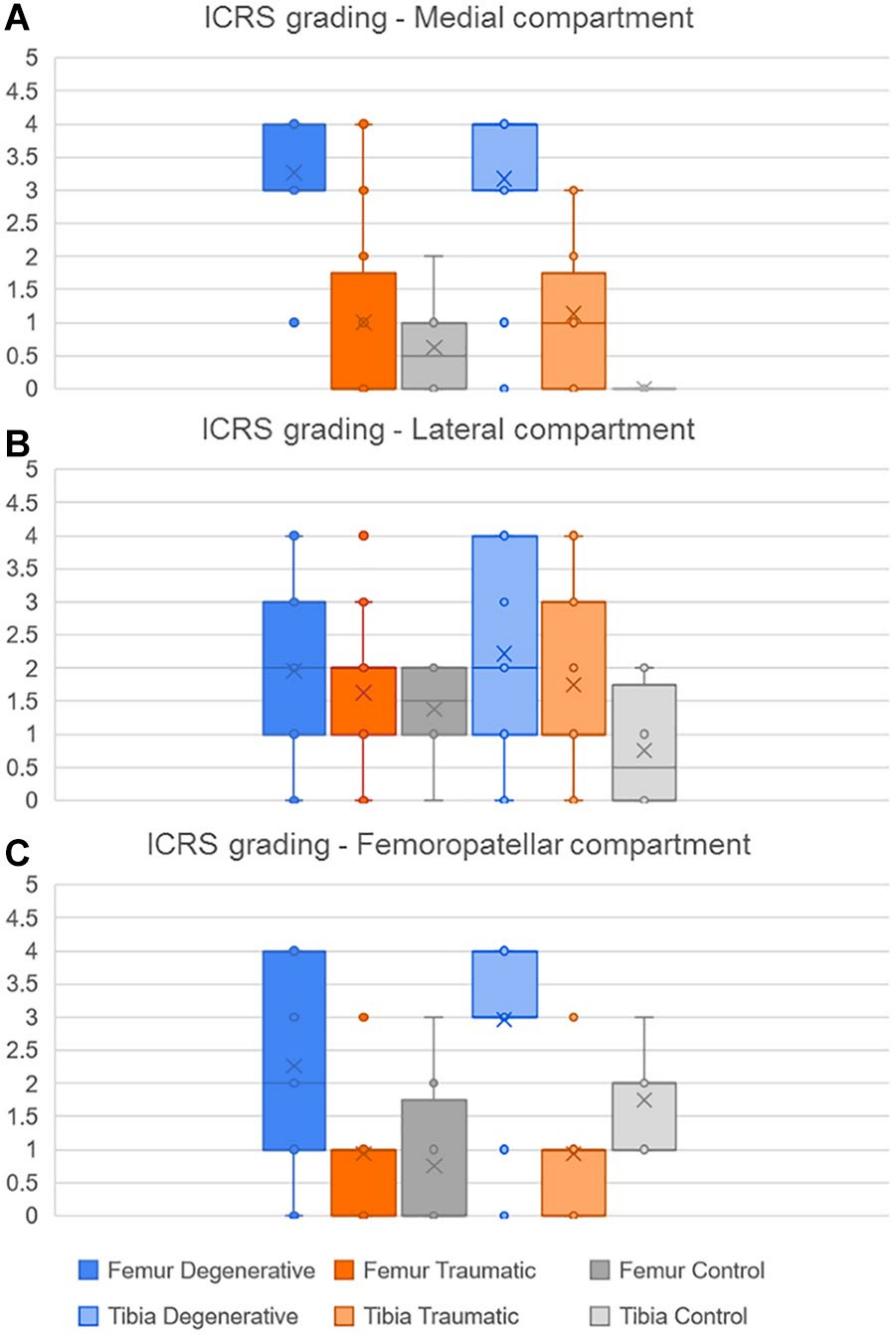
Distribution of the ICRS grading according to bones in each compartment, **(A)** medial, (**B)** lateral and (**C)** femoropatellar, for the 3 groups (degenerative, traumatic and control).

As the ICRS grading is not a binomial result, their average values and standard deviation (SD) for each group are shown instead (**Fig. 12**). The results indicate that in the MC, both femur and tibia had a significant higher grading in the D group compared to T and C (*P* < 0.01). Meanwhile, there were no significant differences of the ICRS grading in the LC bones between the groups. Finally, in the FPC the femoral trochlea of the D group had a significant higher grading (*P* < 0.05) than the T and C groups while in the patella the ICRS values was higher (*P* < 0.05) in D and T when compared to the C group.

#### Cumulative index based on bone conditions

**Figure 13.**
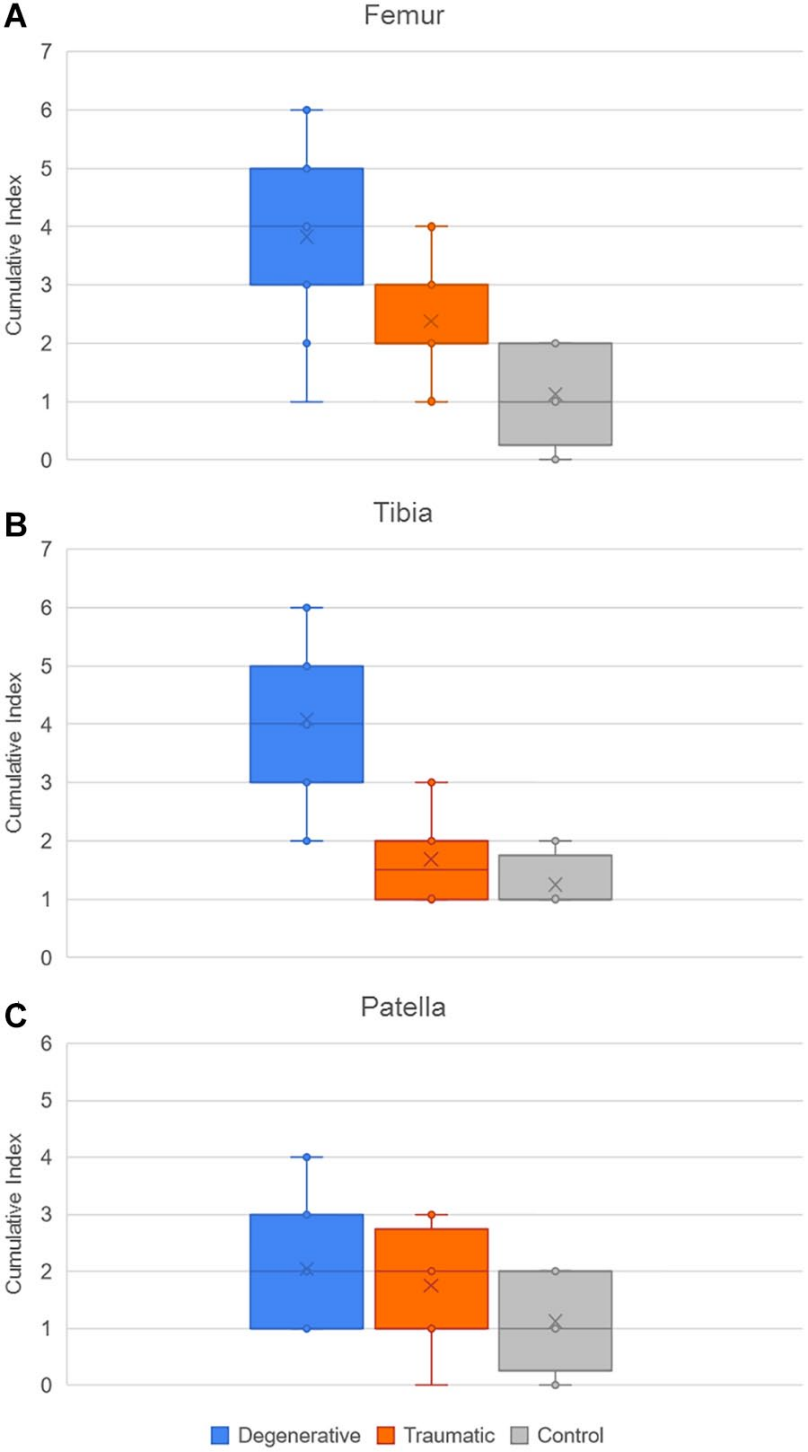
Distribution of cumulative index of pathologies in (**A**) femur, (**B**) tibia, and (**C**) patella for the 3 groups.

For the femur, the cumulative index displayed in **Figure 13** was higher in the degenerative group when compared with the traumatic (*P* < 0.05) and control (*P* < 0.01) groups, while the CI of the traumatic group was higher than the CI in the control 1 (*P <* 0.05). Then, for the tibia, the degenerative group presented a higher index than the traumatic and control groups (*P* < 0.01).

**Figure 14.**
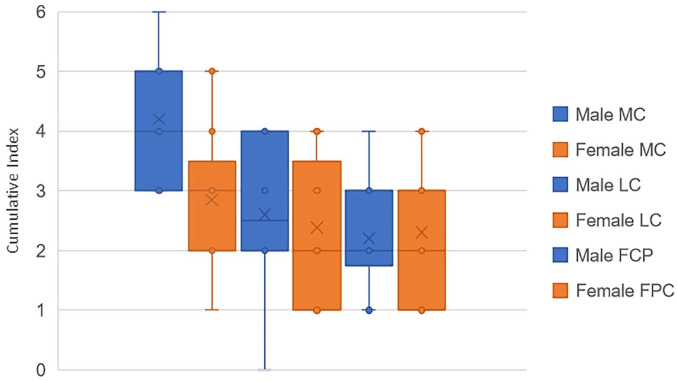
Comparison of cumulative index of pathologies between males (blue) and females (orange) in D group, for the 3 compartments.

The cumulative index has then been investigated more precisely in the D group, to observe the results in the frame of sex and age. **Figure 14** below compares the cumulative index in the 3 different compartments (MC, LC, and FPC), for males and females. No significant differences have been observed between the 2 cohorts. The results according to age will be detailed in the following section.

#### Comparison of different metrics to assess cartilage degeneration

##### CI, Ahlbäck and ICRS grading vs age in the D group

**Figure 15.**
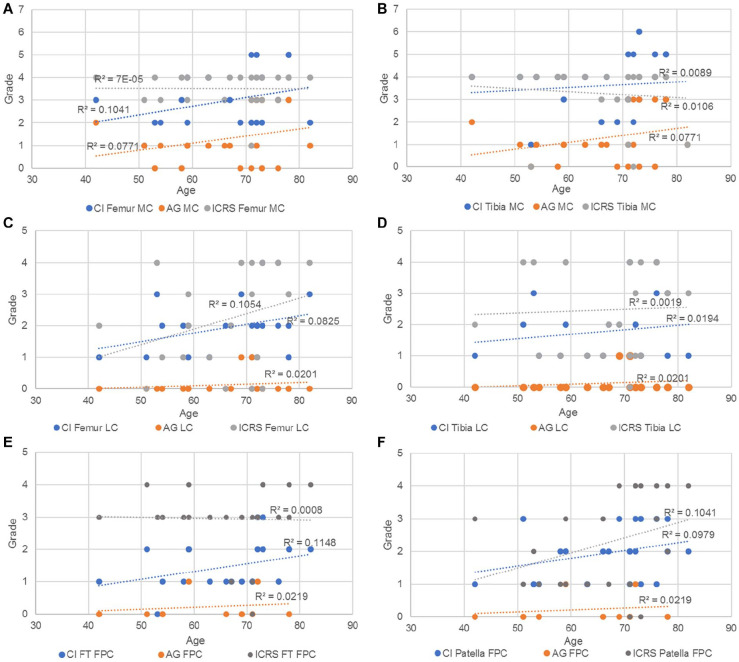
Trendlines and the r^2^ coefficient of the CI, AG and ICRS vs age in (**A**) femur and (**B**) tibia in the MC; (**C**) femur and (**D**) tibia in the LC; (**E**) femoral trochlea and (**F**) patella in the FPC. CI = cumulative index; ICRS = International Cartilage Repair Society; MC = medial compartments; LC = lateral compartments; FPC = femoropatellar compartment.

**Figure 15** shows age dependency in degenerative group (D) for the different grading system: AG, ICRS, and CI. While the CI and AG were positively correlated in all cases, the ICRS grading was positively correlated with age for both femur and tibia in the lateral compartment and for the patella in the FPC. In the rest of the cases, the ICRS was not correlated (a, e) or negatively correlated with age (b).

##### CI and cartilage thickness vs age

**Figure 16.**
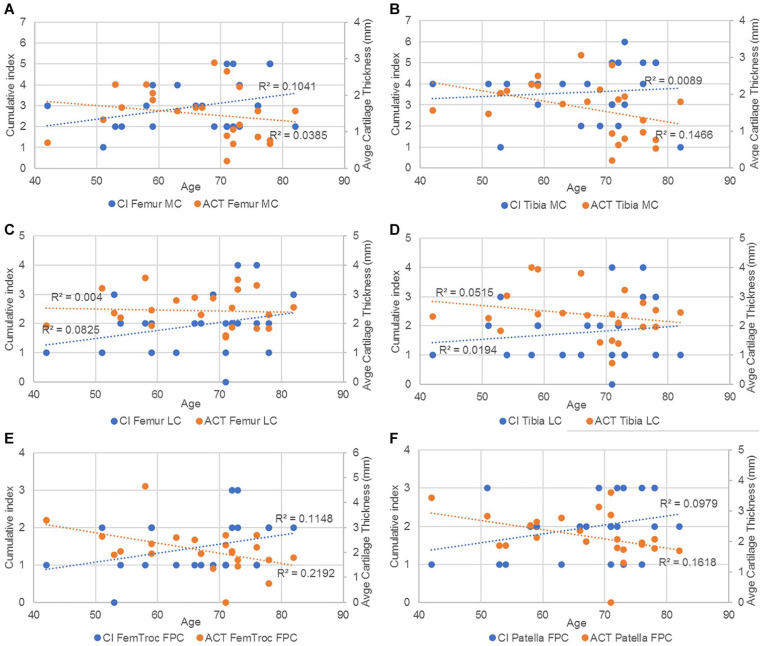
Trendlines and the r^2^ coefficient of CI and average cartilage thickness vs age in (**A**) femur and (**B**) tibia in the MC; (**C**) femur and (**D**) tibia in the LC and (**E**) femoral trochlea and (**F**) patella in the FPC. CI = cumulative index; MC = medial compartments; LC = lateral compartments; FPC = femoropatellar compartment.

For the next comparison (**Figure 16**), the ACT of femur and tibia was considered as the average thickness of the 3 contact points (anterior, middle, and posterior) measured in the medial as well as in the lateral compartment. Meanwhile, for the femoropatellar compartment, the ACT was considered as the average of the 2 contact points (medial and lateral) in the femoral trochlea and of 3 contact points (medial, center, and lateral) in the patella. The results show that the cumulative index was positively correlated with age, while the average thickness of the cartilage was negatively correlated with age in all cases.

##### Summary of statistics

**Table 5** shows the statistical power analysis for the following 2D measurements: CI, ICRS, and cartilage thickness.

**Table 5.** Statistical Power Analysis of 2D Measurements. Sample Size Required for a Power of 80% and a Significance Level at 5%.

	CI_Femur	CI_Tibia	CI_Patella			
**D vs T**	effect size = 1.37	effect size = 2.36	effect size = 0.32			
	Sample size= 9	Sample size= 4	Sample size= 150			
**D vs C**	effect size = 2.72	effect size = 3.11	effect size = 1.08			
	Sample size= 3	Sample size= 3	Sample size= 14			
**T vs C**	effect size = 1.46	effect size = 0.70	effect size = 0.74			
	Sample size= 8	Sample size= 33	Sample size= 29			
	ICRS_Femur_MC	ICRS_Tibia_MC	ICRS_Femur_LC	ICRS_Tibia_LC	ICRS_Femur_FP	ICRS_Patella_FP
**D vs T**	effect size = 2.04	effect size = 1.72	effect size = 0.26	effect size = 0.35	effect size = 1.15	effect size = 0.58
	sample size = 5	sample size = 6	sample size = 222	sample size = 128	sample size = 13	sample size = 48
**D vs C**	effect size = 3.84	effect size = 3.43	effect size = 0.53	effect size = 1.26	effect size = 1.30	effect size = 1.83
	sample size = 2	sample size = 3	sample size = 55	sample size = 11	sample size = 10	sample size = 6
**T vs C**	effect size = 0.33	effect size = 1.51	effect size = 0.26	effect size = 0.94	effect size = 0.17	effect size = 1.50
	sample size = 139	sample size = 8	sample size = 216	sample size = 19	sample size = 530	sample size = 8
	Cart Thick_Avge Fem MC	Cart Thick_Avge Tibia MC				
**D vs T**	effect size = 1.46	effect size = 0.91				
	sample size = 8	sample size = 20				
**D vs C**	effect size = 1.85	effect size = 1.44				
	sample size = 6	sample size = 8				
**T vs C**	effect size = 0.011	effect size = 0.31				
	sample size = 113719	sample size = 157				

CI = cumulative index; ICRS = International Cartilage Repair Society; MC = medial compartments; LC = lateral compartments; FP = femoropatellar.

For the CI, a high effect size is noted for CI femur and tibia, with a sufficient sample size for comparison between D and T groups, and D and C groups. CI patella requires a higher sample size. For the ICRS, D and T groups, and D and C groups can be compared for femoral and tibial MC with a relatively high sample size. Finally, cartilage thickness can be compared between D and T groups, for the femur, and between D and C groups for the femur and tibia.

**Table 6** shows a summary of the statistical analysis made for the CI, ICRS grading, and the ACT between the groups.

**Table 6.** Summary of 2D Measurements Statistics. Hypothesis, Variable, Statistical Test and *P-*Value Are Displayed.

Hypothesis	Variable	Statistical Test	*P-*Value
Difference between D&T groups	CI (Femur)	ANOVA-Bonferroni correction	**0.002**
	CI (Tibia)		**<0.001**
	CI (Patella)		0.99
Difference between D&C groups	CI (Femur)	ANOVA-Bonferroni correction	**<0.001**
	CI (Tibia)		**<0.001**
	CI (Patella)		0.06
Difference between T&C groups	CI (Femur)	ANOVA-Bonferroni correction	**0.033**
	CI (Tibia)		0.98
	CI (Patella)		0.36
Difference between D&T groups	ICRS MC (Femur)	ANOVA-Bonferroni correction	**0.001**
	ICRS MC (Tibia)		**0.001**
	ICRS LC (Femur)		1.00
	ICRS LC (Tibia)		0.85
	ICRS FPC (Femur)		**0.004**
	ICRS FPC (Patella)		0.30
Difference between D&C groups	ICRS MC (Femur)	ANOVA-Bonferroni correction	**0.001**
	ICRS MC (Tibia)		**0.001**
	ICRS LC (Femur)		0.77
	ICRS LC (Tibia)		**0.03**
	ICRS FPC (Femur)		**0.001**
	ICRS FPC (Patella)		**0.001**
Difference between T&C groups	ICRS MC (Femur)	ANOVA-Bonferroni correction	1.00
	ICRS MC (Tibia)		0.08
	ICRS LC (Femur)		1.00
	ICRS LC (Tibia)		0.26
	ICRS FPC (Femur)		1.00
	ICRS FPC (Patella)		**0.014**
Difference between D&T groups	ACT MC (Femur)	ANOVA-Bonferroni correction	***<*0.001**
	ACT MC (Tibia)		**0.006**
	ACT LC (Femur)		0.51
	ACT LC (Tibia)		0.15
	ACT FPC (Femur)		**0.049**
	ACT FPC (Patella)		0.41
Difference between D&C groups	ACT MC (Femur)	ANOVA-Bonferroni correction	***<*0.001**
	ACT MC (Tibia)		**0.005**
	ACT LC (Femur)		0.16
	ACT LC (Tibia)		0.12
	ACT FPC (Femur)		0.28
	ACT FPC (Patella)		1.00
Difference between T&C groups	ACT MC (Femur)	ANOVA-Bonferroni correction	1.00
	ACT MC (Tibia)		1.00
	ACT LC (Femur)		1.00
	ACT LC (Tibia)		1.00
	ACT FPC (Femur)		1.00
	ACT FPC (Patella)		0.68

CI = cumulative index; ICRS = International Cartilage Repair Society; MC = medial compartments; LC = lateral compartments; FPC = femoropatellar compartment; ACT = articular cartilage thickness.

*P*-Values in bold are statistically significant.

The cumulative index in femur and tibia shows a high significance to differentiate D group from the 2 other groups. Similarly, the ICRS shows significant differences between D and C groups for all the compartments of each bone, except for the femoral lateral compartment. Finally, the average cartilage thickness in the medial compartment for femur and tibia shows significant differences between D and the 2 other groups.

### 3D Measurements

The results shown in **Table 7** display the average bone mineral density, as well as radiodensity, volume, and surface from each cartilage after tissue segmentation. The results were calculated for each group (D, T, and C).

**Table 7.** 3D Measurements Results. The Results Show the Average Variable for Each Group (With Standard Deviation Between Parentheses).

	Degenerative	Traumatic	Control
**Bone mineral density (g/cm** ^3^ **)**			
Femur bone	1.32 (1.13)	1.33 (1.14)	1.32 (1.12)
Tibia bone	1.32 (1.13)	1.35 (1.15)	1.29 (1.16)
Patella bone	1.36 (1.12)	1.40 (1.14)	1.41 (1.11)
**Radiodensity (HU)**			
Femur cartilage	85.19 (57.47)	88.67 (49.90)	93.53 (54.37)
Lateral tibia cartilage	87.84 (51.10)	88.69 (44.97)	91.19 (49.21)
Medial tibia cartilage	98.49 (55.92)	93.63 (44.14)	103.79 (52.82)
Patella cartilage	78.36 (50.68)	81.56 (44.97)	99.09 (55.45)
**Volume (mm** ^3^ **)**			
Femur cartilage	17,303 (5,530)	12,460 (2,710)	11,276 (4,505)
Lateral tibia cartilage	2,851 (2,336)	1,100 (439)	1,501 (1,927)
Medial tibia cartilage	1,915 (1,638)	907 (566)	552 (362)
Patella cartilage	2,761 (830)	2,589 (781)	2,703 (705)
**Surface (mm2)**			
Femur cartilage	14,381 (2,636)	12610 (1,496)	11,809 (2,791)
Lateral tibia cartilage	2,435 (1,503)	1,415 (499)	2,073 (2,530)
Medial tibia cartilage	2,016 (1,367)	1,301 (550)	967 (407)
Patella cartilage	2,602 (760)	2,574 (488)	2,495 (390)

HU = hounsfield units.

T group has the highest density of all the bones. D group and C group have similar values for the femur density, while D group has higher density in tibia, and lower in patella. D group has the lowest density in the femoral cartilage, the highest in the patella, and a slightly lower but similar values than C group in both lateral and medial tibia cartilage. D group has the highest cartilage volume for every part. C group has a higher volume than T group for the patella and the lateral tibia. D group has the highest cartilage surface for every part. T group has a higher surface than the C group for the patella and the medial tibia. It can be noted that for the patella, D group has the lowest bone density, the lowest cartilage density, the highest volume, and the highest surface. In general, **Table 6** does not show a significant trend among patient groups due to high interpatient variability; however, patient-specific 3D measurements are used in the machine learning part to enlarge the set of features predicting the patient status.

#### Summary of statistics

**Table 8** shows a summary of the statistical analysis made for the BMD, cartilage radiodensity, and cartilage volume between the groups.

**Table 8.** Summary of 3D Measurements Statistics. Hypothesis, Variable, Statistical Test and *P-*Value Are Displayed.

Hypothesis	Variable	Statistical Test	*P-*value
Difference between D&T groups	BMD (femur)	ANOVA-Bonferroni correction	**0.014**
	BMD (tibia)		***<*0.001**
	BMD (patella)		***<*0.001**
Difference between D&C groups	BMD (femur)	ANOVA-Bonferroni correction	0.82
	BMD (tibia)		1.00
	BMD (patella)		**0.002**
Difference between T&C groups	BMD (femur)	ANOVA-Bonferroni correction	0.72
	BMD (tibia)		0.078
	BMD (patella)		1.00
Difference between D&T groups	CD (femur)	ANOVA-Bonferroni correction	1.00
	CD (tibia)		1.00
	CD (patella)		1.00
Difference between D&C groups	CD (femur)	ANOVA-Bonferroni correction	0.43
	CD (tibia)		1.00
	CD (patella)		0.10
Difference between T&C groups	CD (femur)	ANOVA-Bonferroni correction	0.38
	CD (tibia)		1.00
	CD (patella)		0.11
Difference between D&T groups	CV (femur)	ANOVA-Bonferroni correction	***<*0.001**
	CV (tibia)		0.13
	CV (patella)		0.73
Difference between D&C groups	CV (femur)	ANOVA-Bonferroni correction	***<*0.001**
	CV (tibia)		**0.027**
	CV (patella)		1.00
Difference between T&C groups	CV (femur)	ANOVA-Bonferroni correction	1.00
	CV (tibia)		0.93
	CV (patella)		1.00

BMD = bone mineral density; CD = cartilage density; CV= cartilage volume.

*P*-Values in bold are statistically significant.

#### CI and radiodensity vs age

**Figure 17.**
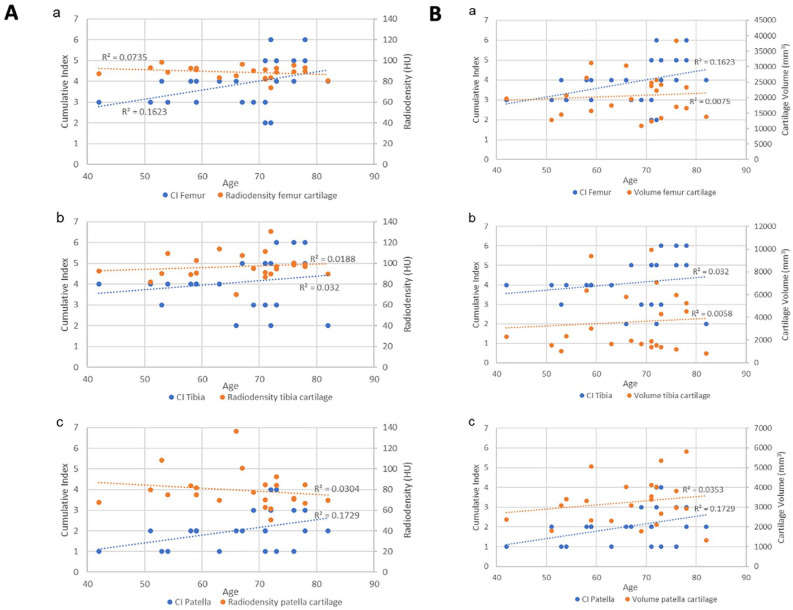
**(A**) Trendlines and the r^2^ coefficient of the CI and the cartilage radiodensity of the (a) femur, (b) tibia, and (c) patella. (**B**) Trendlines and the r^2^ coefficient of the CI and the cartilage volume of the (a) femur, (b) tibia, and (c) patella. CI = cumulative index.

After comparison with other 2D metrics, the cumulative index was compared with the cartilage radiodensity (**Fig. 17A**) and the cartilage volume (**Fig. 17B**) and plotted against the age of those patients who belonged to the D group.

The results show a higher correlation of the cumulative index in very case compared to the cartilage radiodensity and the cartilage volume.

### Machine Learning

**Table 9** shows all the results of the ML analysis. The best accuracy results are 89.4, which is obtained with RF using the whole feature set and the 2D measurements feature set. F1 score is high for the degenerative patients (always around 90%), while it is slightly lower for the other 2 groups. These F1 scores are due to the higher number of degenerative patients but also demonstrate that with the selected features, it is efficient to classify patients with degenerative cartilage using RF and GB. The best metrics for the classification of control subjects are obtained with the 2D feature set, while all 96 features give the best F1 score for classifying traumatic patients using RF. In terms of accuracy, good results are obtained with MRI, bone, and cartilage feature selections, especially with the RF algorithm, while 3D selection is the worst, with a maximum of 76.6 accuracy with GB. With RF, the bone features give better results in all 3 classes compared to the cartilage feature set. Using GB and cartilage feature set, only control subjects are classified with higher metrics. We can state that RF is the most efficient of the 2 tree-based algorithms.

**Table 9.** Classification Metrics (Recall [Re] Precision (Pr) and F1 [%]) for the 2 Different Tree-Based ML Algorithms and the Seven Different Features Selections (Degenerative [D]—Traumatic [T]—Control [C]).

Features Selection	Alg.	Acc.	Re [D]	Pr [D]	F1 [D]	Re [T]	Pr [T]	F1 [T]	Re [C]	Pr [C]	F1 [C]
Tot Feat [96]	RF	89.4	95.9	92.0	93.9	93.3	82.4	87.5	62.5	100	76.9
	GB	87.2	91.7	95.7	93.6	93.3	73.7	82.4	62.5	100	76.9
2D Feat [78]	RF	89.4	91.7	91.7	91.7	86.7	86.7	86.7	87.5	87.5	87.5
	GB	87.2	91.7	91.7	91.7	80.0	80.0	80.0	87.5	87.5	87.5
3D Feat [18]	RF	74.5	83.3	83.3	83.3	66.7	66.7	66.7	62.5	62.5	62.5
	GB	76.6	87.5	84.0	85.7	66.7	76.9	58.8	62.5	55.6	58.8
CT-Scat Feat [26]	RF	80.9	91.7	95.7	93.6	66.7	71.4	69.0	75.0	60.0	66.7
	GB	74.5	87.5	84.0	85.7	60.0	75.0	66.7	62.5	50.0	55.6
MRI Feat [52]	RF	87.2	95.8	95.8	95.8	87.6	76.5	81.2	62.5	83.3	71.4
	GB	87.2	91.7	88.0	89.8	80.0	85.7	82.8	87.5	87.5	87.5
Bone Feat [50]	RF	85.1	91.7	91.7	91.7	86.7	76.5	81.2	62.5	83.3	71.4
	GB	76.6	87.5	95.5	91.3	73.3	64.7	68.8	50.0	50.0	50.0
Cartilage Feat [26]	RF	83.0	91.7	88.0	89.8	73.3	73.3	73.3	75.0	85.7	80.0
	GB	83.0	91.7	88.0	89.8	67.7	76.9	71.4	87.5	77.8	82.4

ML = machine learning; RF = random forest; GB = gradient boosting; MRI = magnetic resonance imaging.

**Tables 10** and **11** show the 12 most important features and the percentage of importance of all the different feature groups for the RF classification ML model using the 96 total features as input. This model was selected for the feature importance analysis because it can be considered the most significant in terms of accuracy (89.4 is the highest). It allows for a complete overview of the full set of features and their respective importance in the classification process. The highest importance is attributed to 2 features from the 3D collection (the volume of the tibialis cartilage lateralis and medialis). The 3D features set contribute 33% of importance despite being only 18 compared to the 78 2D features. The cartilage set of features has higher importance compared to the bones. In contrast, the CT scan features contribute to only 14.39% of the importance having only 2 of them in the first 12 most important features (lateral and medium osteophytes).

**Table 10.** 12 Most Important Features [%] for the RF Classification Model With 96 Tot Features.

TibCartLatVOL [mm3]	4,804
TibCartMedVOL [mm3]	4,631
CT Lat Osteophytes	4,594
MRI Med Cart Thick FEM [mm]—Med	4,262
MRI Med Menisc Pathol	3,909
MRI Lat Osteophytes	3,805
FemCartVOL [mm3]	3,644
TibCartLatSTD	2,543
MRI Lat Cart Thick FEM [mm]—Ant	2,491
MRI Med Cart Thick FEM [mm]—Post	2,402
MRI Lat Cart Thick TIB [mm]—Med	2,352
CT Med Osteophytes	2,343

RF = random forest; CT = computed tomography; MRI = magnetic resonance imaging.

**Table 11.** Importance of the Groups of Features [%] for the RF Classification Model With 96 Tot Features.

2D	66.08%
MRI (part of 2D features)	51.69%
CT (part of 2D features)	14.39%
3D	33.12%
BONE (from CT and MRI)	28.29%
CARTILAGE (from MRI)	37.79%

RF = random forest; MRI = magnetic resonance imaging; CT = computed tomography.

## Discussion

This work developed a methodology to evaluate cartilage degeneration. It uses a multimodal image approach to segment, and 3D model bones and cartilages from the knee area. Indeed, the MRI provides information about pathologies, morphology of the cartilages, and a geometric representation of the tissue damage, while CT data present a good overview of bone pathologies, especially in boundary regions. The combination of both imaging techniques gives a 3D representation of the knee, and additional information about bone and cartilage. These data overview makes the definition of 96 features possible, which demonstrated various levels of significance with regard to contribution toward the cartilage quality evaluation.

From the 2D measurements, the first parameters shown in the results are those which better testify to degeneration on the D group such as the presence of cysts, osteophytes, and meniscal pathology in the MC when compared to the T and C groups. This agrees with other biomechanical evidence^45^ which suggests that the medial tibiofemoral joint reaction forces are greater than the lateral ones during gait and therefore the degeneration of the cartilage and the surrounding tissue would be more evidently seen on the medial side. Meanwhile, the T group demonstrated greater incidence of subchondral edema within the femoropatellar compartment when compared to the other 2 groups, making this an interesting and useful indicator, for example, in the development of protective elements to avoid injury. The cumulative index, which is based on the bone condition, indicates statistically that the degenerative patients present more pathologies in femur and tibia than the other patients. These results were expected, as the degeneration of the knee is known to affect the condition of both bones and cartilage, as confirmed by the ICRS values, which showed a significantly worse cartilage condition for the D group within the medial and femoropatellar compartments. Consequently, to assess the accuracy/utility of the cumulative index as an indicator of cartilage degeneration, the CI was subject to comparison with other 2D metrics only within the D group in the frame of age, since males and females did not show significant differences in any of the 3 compartments. Therefore, when compared to the AG and the ICRS for the different compartments the CI showed a regular trendline suggesting a higher index with aging of the degenerative patients. In some cases, the *r*^2^ indicated a better fit for the CI than the other metrics as in the femur in the medial and FPC. Later, when compared with the average cartilage thickness, the cumulative index presented a regular trend as well as the thickness, where the older the patient, the thinner the cartilage, and the higher the index. In summary, the cumulative index based only on the bone condition shows a way to differentiate the groups, and it is strengthened by the statistical power analysis. Then, when compared against other metrics, the general low R^2^ values of the index are an indication of the high interpatient variability, which (despite the small sample size) was expected based on our hypothesis of needing higher sensitivity in the assessment of cartilage degeneration toward improving patient-specific profile and subsequently, treatment.

The fact that degeneration is mainly located in the same areas explain why the D group is easier to discriminate. The T group shows less homogeneous results, where only subchondral edemas helped to differentiate them. Trauma affects a specific region that varies for each patient; therefore, it is harder to find any similar trends according to which bone or cartilage is studied.

The BMD calculated for the femur, and tibia bones do not discriminate the cartilage condition, probably because we calculated the overall BMD over the entire scanned bone segment without focusing on the boundary Bone/Cartilage. Moreover, the results on the patella bone show higher BMD in healthy and traumatic individuals. The likeliest reason is that in this case, we have a complete and small bone volume, and a limited HU variability.^46^ The density calculated on the entire cartilage volume is also not a significant discriminator between degenerative, traumatic, and healthy conditions. However, a trend of decreased density in degenerative cartilage can be seen after eroding the cartilage mask and filtering the HU distribution. After this process, the differences between healthy and pathological conditions were more evident. This is in line with a known behavior regarding tissue density and pathologies. It has been shown that people suffering from rheumatoid arthritis present lower muscle density associated with joint destruction.^47^ Thus, people suffering from degenerative cartilage can present similarly a lower cartilage density associated to this damaged joint. For these reasons, we wanted to examine the cartilage density and cartilage volume alongside the bone CI, on the D group. The study of CI and cartilage density regarding age (**Fig. 14**) reveals that for each cartilage, the cartilage density remains similar through age, whereas the CI shows an increasing trend. The same behavior is observed regarding the volume, which does not change much with age compared to CI. The low R^2^ (R^2^ < 0.04) in both volumes and radiodensity metrics, lead us to believe that even though those parameters should be considered for group classification, they do not provide sensitive enough information to thoroughly assess cartilage degeneration. One reason for that is the lack of objective automatic methods for segmentation,^48^ leading to a bias or a lack of accuracy for 3D segmentation, that influences the data results. Future studies with more advanced segmentation techniques should confirm this outcome. For now, the best indicator identified in our study to quantify cartilage degeneration is the bone cumulative index.

ML results of **Table 4** underline significant recall and precision, as well as F1, especially on the classification of degenerative patients, reaching a maximum recall value of 95.9 using the total and MRI feature selections, having almost 90% accuracy. The use of all the 96 features gives the best classification metrics and allows a complete feature importance analysis which gives new significant hints for studying the degeneration condition of the knee cartilage.

Noteworthy are the results obtained with the single Bone and Cartilage feature selections. While a good accuracy value is expected for the latter as we are classifying subjects relative to their cartilage status, the classification metrics obtained with the bone selection are of high impact. Cartilage status is highly dependent on the bone’s condition, as has been demonstrated by Cai *et al.*,^49^ which observed changes in the subchondral bone with OA progression. Moreover, Bonakdari *et al.*^50^ recently used bone features to predict cartilage volume loss obtaining a correlation coefficient of more than 0.78. Similarly, we demonstrated that bone has high importance in the classification process. Still, if combined with cartilage and 3D features, the metrics significantly increase, indicating that with the contribution of all these sets of features, a more in-depth view of the knee cartilage status can be given. If we consider 3D features or cartilage feature sets alone for the classification process, the metrics are not significant due also to the limited number. But, looking at the results in Tables 5 and **6**, they assume a significant relevance if we consider the whole complete set (the 2 most relevant features, volume of the tibialis cartilage lateralis and medialis, are from the novel 3D group). 3D features contribute one-third of the importance despite the limited number. At the same time, they give the lowest accuracy of 74.5 if considered the only input to the tree-based algorithms. The 26 cartilage features alone can give a decent 83% accuracy but, if taken together with the other 70, contribute to the classification for almost 40% of total importance. We can conclude that the complete set of features gives the best input for future developments of this study: all the 96 bone, cartilage, and 3D features together could be used to develop new clinical solutions like the design of a patient-specific cartilage status profile which will help the clinicians and the researchers in an easier and objective classification of the cartilage status and an evaluation of the degeneration level. This novel methodology, combining 2D and 3D measurements, is of interest to assess cartilage quality. By designing indexes of pathology and combining it with other parameters such as radiodensity, it is possible to categorize cartilage into a group condition. This preliminary study should be pursued with a larger range of subjects to ensure its efficiency.

### Limitations

The 2D assessment through CT and MRI is normally performed by 1 person and can be sometimes difficult to assess some pathology because of the image quality or the subjectivity of the researcher and this situation might affect the evaluation of the bone and cartilage. Likewise, the 3D segmentation process is quite long and mostly manual. Despite multiple adjustments and cross-verification, some inaccuracies can remain, which can affect the 3D pipeline, therefore the density, volume, and surface values. An improvement of the segmentation workflow, using a semi-automatic or fully automatic method, would help to get more accurate values.

The number of subjects in each group, especially in the C group, remains small. Therefore, the statistical results must be observed with caution, and the preliminary trend observed to differentiate the groups has to be confirmed with a larger sample of participants.

The bone 3D measurements suffered from a high variability because we are studying the whole bone instead of regions of interest (boundaries around the cartilage). It has been done this way to avoid partial volume effect and image artifacts. However, it also results in a less precise way to evaluate bones.

ML analysis also presents some limitations. The number of subjects is not particularly high; this could affect the classification performances and a partial overfitting may occur in some models. Moreover, the multi-class approach can significantly decrease the classification metrics: for future work, a binary classification option can be performed to study the prediction potential of the features to distinguish degenerative patients from all the others. A higher number of control subjects could also potentially be the starting point for a binary classification between degenerative vs healthy or traumatic vs healthy subjects.

## Conclusion

We developed a cartilage segmentation and 3D modeling procedure that can be used as benchmark for 3D bioprinting design and to advance cartilage assessment. Based on a cumulative index of bone properties (CI), we demonstrate the importance of bone condition and the sensitivity of these measurements on medial and femoropatellar compartments. Moreover, we show that a combination of 2D radiological measurements and 3D measurements revealed potential biomarkers of cartilage degeneration, especially from medial femur.

This work is a first step toward a patient-specific cartilage profile based on the combination of CT and MRI datasets. This could be crucial for improving cartilage assessment. Indeed, when evaluating patients with knee pain either following trauma or with acute or chronic illness, the patient’s symptoms are always the cornerstone in the treatment decision, whether medical or surgical. Following plain x-ray, a CT scan and most often also MR scan are the best tools in elucidating the interior of the knee joint. The CT scan is both easy to get and fast to execute but uses ionizing radiation. It reveals, however, best all the bony structures and injuries. It may also give some clues about the bone marrow and surrounding soft tissues. The MR, however, is the best examination to evaluate the status of both the cartilage and the ligaments. The drawback is both the long time until it can be executed and long running time which can sometimes be impossible in patients with severe pain. When merged, these 2 examinations give the most superior evaluation ever for the knee joint and should always be chosen prior to invasive arthroscopy. Our study shows the feasibility of extending the cartilage assessment using existing and new parameters from both image modalities.

## Supplemental Material

sj-docx-1-car-10.1177_19476035221144746 – Supplemental material for Toward New Assessment of Knee Cartilage Degeneration Click here for additional data file. Supplemental material, sj-docx-1-car-10.1177_19476035221144746 for Toward New Assessment of Knee Cartilage Degeneration by Romain Aubonnet, Jorgelina Ramos, Marco Recenti, Deborah Jacob, Federica Ciliberti, Lorena Guerrini, Magnus K. Gislason, Olafur Sigurjonsson, Mariella Tsirilaki, Halldór Jónsson jr and Paolo Gargiulo in CARTILAGE
