# Supplementary material for: Toward New Assessment of Knee Cartilage Degeneration
Source: Cartilage. 2022 Dec 21;14(3):351–74. doi: 10.1177/19476035221144746 (PMC10601563; doi:10.1177/19476035221144746)
Supplement: sj-docx-1-car-10.1177_19476035221144746 – Supplemental material for Toward New Assessment of Knee Cartilage Degeneration [file sj-docx-1-car-10.1177_19476035221144746.docx]

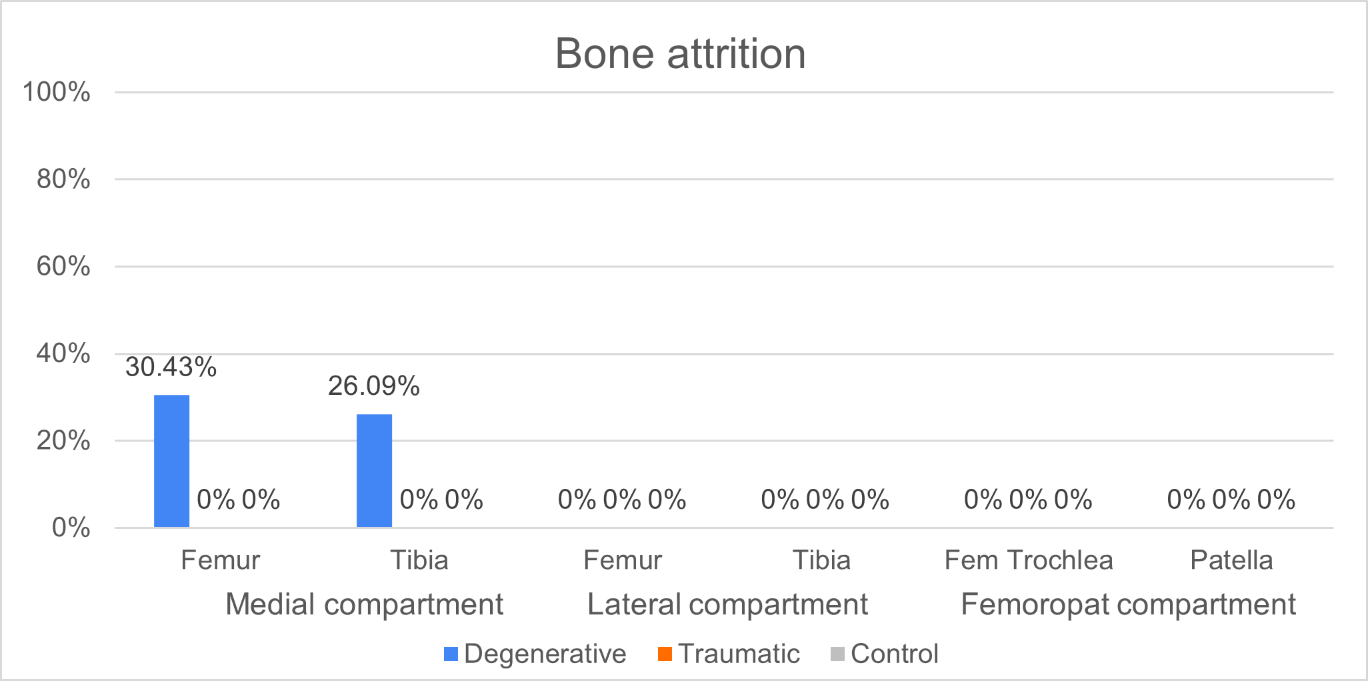


**Figure S1 – Bone attrition distribution. % of patients from each group with bone attrition according to the bone in each compartment.**

**
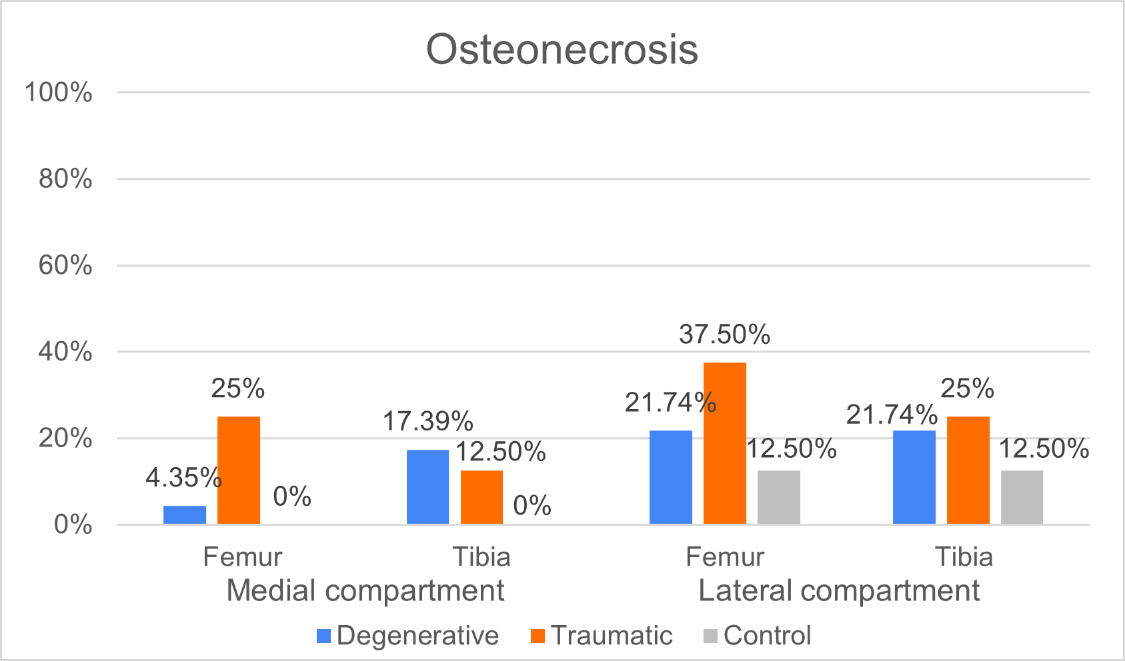
**

**Figure S2 – Osteonecrosis distribution. % of patients from each group with osteonecrosis according to the bone in each compartment.**

**
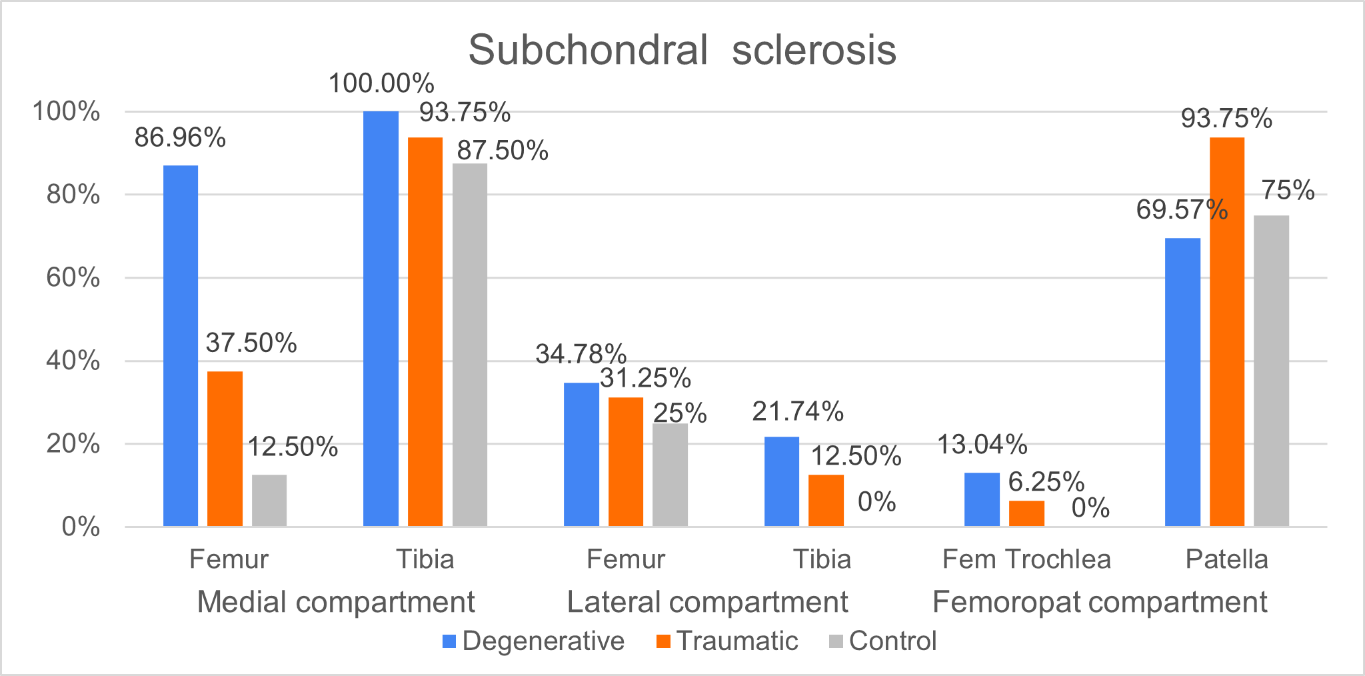
**

**Figure S3 – Subchondral sclerosis distribution. % of patients from each group with subchondral sclerosis according to the bone in each compartment.**
